# Acoustic emissions of *Sorex unguiculatus* (Mammalia: Soricidae): Assessing the echo‐based orientation hypothesis

**DOI:** 10.1002/ece3.4930

**Published:** 2019-02-15

**Authors:** Lida Sanchez, Satoshi D. Ohdachi, Atsushi Kawahara, Lazaro M. Echenique‐Diaz, Shinichiro Maruyama, Masakado Kawata

**Affiliations:** ^1^ Graduate School of Life Sciences Tohoku University Sendai Japan; ^2^ Institute of Low‐Temperature Science Hokkaido University Sapporo Japan; ^3^ Hokkaido Regional Environment Office Ministry of Environment Sapporo Japan; ^4^ Miyagi University of Education Sendai Japan

**Keywords:** echolocation, habitat acoustics, the long‐clawed shrew, vocal behavior

## Abstract

Shrew species have been proposed to utilize an echo‐based orientation system to obtain additional acoustic information while surveying their environments. This system has been supported by changes in vocal emission rates when shrews encounter different habitats of varying complexity, although detailed acoustic features in this system have not been reported. In this study, behavioral experiments were conducted using the long‐clawed shrew (*Sorex unguiculatus*) to assess this orientation system. Three experimental conditions were set, two of which contained obstacles. Short‐click, noisy, and different types of tonal calls in the audible‐to‐ultrasonic frequency range were recorded under all experimental conditions. The results indicated that shrews emit calls more frequently when they are facing obstacles or exploring the experimental environment. Shrews emitted clicks and several different types of tonal calls while exploring, and modified the use of different types of calls for varying behavior. Furthermore, shrews modified the dominant frequency and duration of squeak calls for different types of obstacles, that is, plants and acrylic barriers. The vocalizations emitted at short inter‐pulse intervals could not be observed when shrews approached these obstacles. These results are consistent with the echo‐based orientation hypothesis according to which shrews use a simple echo‐orientation system to obtain information from their surrounding environments, although further studies are needed to confirm this hypothesis.

## 1 INTRODUCTION

Animals develop the abilities of habitat assessment and foraging through different sensory capabilities (Benoit‐Bird & Au, 2009; Furlonger, Dewar, & Fenton, 1987; Gould, McShea, & Grand, 1993; Schnitzler, Kalko, Kaipf, & Grinnell, 1994; White, Day, Butler, & Martin, 2007). Several bat and dolphin species accomplish these activities through the emission and reception of sounds, also known as echolocation (Griffin, 1958). These mammals avoid obstacles and receive detailed information about their prey through different acoustic cues (Simmons et al., 1983). These species can actively control the spectral features (i.e., peak frequencies, frequency slope, and frequency range) and temporal scale (i.e., call duration and inter‐pulse interval) of their vocalizations in order to extract information from their environments (Moore, 1988; Neuweiler, 1984). Echolocation has also been suggested to exist in other animal groups, such as oilbirds and shrews (Forsman & Malmquist, 1988; Griffin, 1953).

A great diversity of vocalizations have been described within the shrew's repertoire (Schneiderová, 2014; Volodin, Zaytseva, Ilchenko, & Volodina, 2015; Zsebők, Czabán, Farkas, Siemers, & Merten, 2015). Some of these vocalizations have been linked to the shrew's exploratory behavior, such as click calls. These calls have been suggested as the main component involved in the shrew's echolocation (Buchler, 1976; Forsman & Malmquist, 1988). However, recent studies recording full spectrum vocalizations of *Sorex araneus* and *Crocidura russula* uncovered tonal vocalizations related to this exploratory behavior. Individuals of these species increased the number of their vocalizations with increases in the level of complexity of their environments. From playback experiments conducted using these recorded calls, authors found differences in the returning echoes from different items present in the shrews’ environments, suggesting shrews might extract information from these objects to orientate themselves (Siemers, Schauermann, Turni, & Merten, 2009). Despite the similarities of this behavior with those developed by other echolocating species (Falk, Jakobsen, Surlykke, & Moss, 2014), the researchers described this behavior as echo‐based orientation instead of attributing it to echolocation. They suggest this system may be simpler than echolocation, thus giving shrews a coarse image of their surroundings.

However, these studies have primarily referred to the behavioral responses of shrews without providing a more detailed acoustical description of their vocalizations in an exploratory context. It is unclear whether shrews can adjust the acoustic parameters of their calls in order to extract information from their environments, as occurs with other echolocating species. Extensive studies have been conducted on echolocating bats and cetacean species, showing general and particular features of this orientation system (Au, 2012; Thomas & Jalili, 2004). However, accurate attribution of echolocation or echo‐based orientation in shrews is not completely clear since information concerning various aspects of the shrew's vocal behavior and acoustic features of their vocalizations during exploration is lacking.

In this study, we assessed the echo‐based orientation hypothesis with a focus on vocal behavior. The vocal response itself could suggest the use of echo‐based orientation by shrews if modifications in their calls are found when they need to orient themselves in a new environment. We characterized the sounds emitted by long‐clawed shrews (*Sorex unguiculatus*) when encountering obstacles under experimental conditions. We examined whether individuals adjusted the number of calls and call parameters (e.g., type of call, duration, and dominant frequency) under different conditions. We also evaluated whether call parameters and rates were affected by specific behaviors of these animals within each experimental condition.

## 2 MATERIALS AND METHODS

### 2.1 Captured individuals and animal care

We captured five *S. unguiculatus* in October 2016 from Kenbokki Island, Eastern Hokkaido Prefecture, Japan, using pitfall traps that were opened at sunset and closed after sunrise. The traps were checked every 2 hr. The animals were housed in separated plastic cages containing hay in a laboratory at the Hokkaido University, Sapporo City, Japan. Water and food were supplied ad libitum. The food provided included canned boiled crickets, raw minced pork meat, silkworm pupae, as well as live and cooked canned mealworms. For animal care and breeding procedures, we followed the guidelines of the Institutional Animal Care and Use Committee of the Tohoku University (Permission number 2017LSA‐024).

### 2.2 Recording acoustic vocalizations and behavioral experiments

The shrews were subjected to three experimental conditions for behavioral experiments. These three conditions were repeated twice for each animal, except one shrew, which could not be tested under soft‐barrier conditions. During these experiments, an individual shrew was removed from its resident cage and placed into a larger plastic cage (LWH, 65.5 × 33 × 37 cm) for 2 hr (Supporting Information Figure S1). This relocation to a new cage for a limited time to conduct these experiments instigated an increased vocal response, as previously reported (Buchler, 1976). Each animal was numbered in order to place them under these experimental conditions randomly.

The experimental cage contained a 3‐cm‐thick layer of sand to reduce noise, a wooden box for shelter with a single entrance to allow the animals to rest, and two dishes containing either food or water. To avoid any residual odor from the previously caged animal, the soil, the wooden shelter (i.e., a wooden box for each), and the food and water dishes were changed each time a new shrew was introduced into the cage. The food dish and shelter box were placed in the experimental cage immediately before initiating the experiment.

The experimental conditions consisted of two different obstacle arrangements as well as a control condition. In the control condition, only the wooden box and dishes were present (Supporting Information Figure S1a). The other two experimental conditions presented these afore mentioned items and obstacles located in the middle of the cage, defined as hard‐barrier and soft‐barrier. For the former, we placed acrylic plates equidistant from each other and the walls of the plastic cage (Supporting Information Figure S1b). The height of these acrylic plates was almost the same as that of the experimental cage, forcing the shrews to walk among them, finding a path throughout the enclosure. The second condition included two plastic plants in the same locations as the acrylic plates (Supporting Information Figure S1c). These plants most likely provided a soft and low amplitude echo (Yovel, Stilz, Franz, Boonman, & Schnitzler, 2009), opposed to a possibly higher amplitude echo from the acrylic plates. Shrews crawled through the plants most of the time, instead of walking around them. These conditions allowed us to determine whether acoustic information was involved in their orientation within their surroundings.

All experiments were conducted at night; animal behavior was recorded using a night vision video camera (HX‐A1H; Panasonic, Japan) coupled with infrared light. Acoustic vocalizations were recorded using an SMM‐U1 omnidirectional microphone (frequency response between 5–80 kHz; Wildlife Acoustics, Maynard, MA, USA) attached to an SM4BAT FS automated recording unit (Wildlife Acoustics) programed to record during the 2 hr of each experiment. The microphone was calibrated at the beginning of each experimental session using an ultrasonic calibrator (Wildlife Acoustics) to ensure its accurate response. Vocalizations were sampled at a frequency of 256 kHz, 16 bits.

The acoustic recorder was triggered only when an 18‐dB threshold was surpassed. The recorder continued recording 15 s after the vocalizations ceased. The video camera and the microphone were attached to a tripod and placed on top in the center of the experimental cage, approximately 12 cm from the animals. The entire cage, including the camera and microphone setup, was covered with acoustic foam to reduce any background noise. To accurately attribute shrew behavior to the emitted vocalizations, the recorded audio files were analyzed in combination with the videos. Also, the sound of the shrews eating (i.e., a distinctive chewing sound) enabled a more precise synchronization of the video and audio into a single file than that obtained using timestamps in the files.

Placing animals in the experimental cage induced several different behaviors. These behaviors then were classified as follows: (a) On the food plate (OFP), shrews stood on the food plates or approached them; (b) Shelter (SH), shrews moved in and out of the shelter or stood above it; (c) Facing obstacles (FO), shrews stopped or momentarily rested their front limbs on the acrylic plates and plants or crawled over/through the plants; and (d) Exploring (EXP), shrews stood on the cage walls and walked quickly around the cage without any specific direction. If shrews emit calls for orientation purposes, they should call more frequently when they are probing their environment, that is, during FO and EXP behaviors. The sounds emitted while the animals were eating and digging were excluded from the analysis. In the control condition, the shrews could not exhibit FO behavior since no obstacles were placed in the experimental cage.

During the 2 hr for each experiment, when the animals moved throughout the experimental cage, the duration of this movement was recorded (i.e., minutes) and defined as active. When animals remained inside the shelter, they were considered to be resting. We counted the number of times each animal demonstrated each of the behaviors listed above. These behaviors were annotated even when no vocalizations were recorded. The sound files were grouped according to behavioral categories for further acoustic analyses.

### 2.3 Analyses of acoustic vocalizations

The R packages seewave (Sueur, Aubin, & Simonis, 2008) and warbleR (Araya‐Salas, & Smith‐Vidaurre, 2017) were used to detect, characterize, and represent the recorded vocalizations. The threshold used to detect vocalizations within the sound files varied between 12 and 20 dB, depending on the signal‐to‐noise ratio of each file.

**Figure 1:**
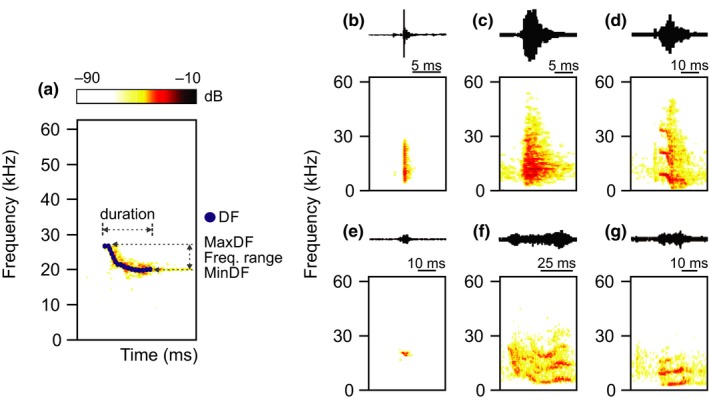
Spectrograms and waveforms of the vocalizations recorded for *Sorex unguiculatus*. (a) Call variables measured for these vocalizations included call duration, dominant frequency (DF), maximum dominant frequency (MaxDF), minimum dominant frequency (MinDF), and frequency range (Freq. range). The call types were (b) click, (c) noisy, and tonal calls, which included (d) chirp, (e) short scream, (f) squeak, and (g) twitter

The acoustic variables measured from the selected calls included call duration (millisecond [ms]), dominant frequency (kilohertz [kHz]; reported as mean value), minimum dominant frequency (kHz), maximum dominant frequency (kHz), and frequency range (kHz; calculated as the difference between the minimum and maximum dominant frequencies; Figure 1a). The following settings were used on the spectrograms: FFT length 256, Hanning window, and 95% overlap. For all calls, the intensity threshold for measuring these variables was 10 dB. Following a visual inspection of call spectrograms, the recorded vocalizations were classified into the following three classes: click (short broadband; Figure 1b), noisy (lacking fundamental frequency; Figure 1c), and tonal (a well‐defined frequency contour; Figure 1d–g). These classifications were defined according to the criteria proposed by Schneiderová (2014). The recorded tonal calls were further classified based on the criteria above and by that specified by Volodin et al. (2015) via observation of the spectral patterns of the tonal calls within the spectrograms. Tonal calls were classified into the following four categories: chirp (frequency‐modulated calls presenting different spectral patterns; Figure 1d), short scream (short calls weakly modulated in frequency, commonly exhibiting a reverse U shape; Figure 1e), squeak (long calls with deeply modulated frequency; Figure 1f), and twitter (low‐frequency multiharmonic calls with variable durations and slightly modulated frequencies; Figure 1g).

### 2.4 Statistical analyses

Statistical analyses were performed using the R language (R Core Team, 2000). The effect of the experimental conditions on the number, duration, and dominant frequency of the calls was tested using regression analysis and generalized linear mixed models. In the model, the experimental conditions were designated as a fixed effect and individuals as random effects. For examining the effect of the experimental conditions on the number of calls, the Poisson link function was used. For the effects on duration and dominant frequency of the calls, values were log‐transformed. We also tested the effects of behavioral categories on the number of vocalizations, call duration, and dominant frequency. These were performed considering possible effects on the vocal behavior of shrews according to the experimental condition. The behavioral categories were designated as fixed effects and the number of individuals as random effects. This analysis was conducted separately for tonal and click vocalizations. A similar analysis was conducted to test the effect of behavioral categories on the number of tonal type vocalizations, their call durations, and mean dominant frequencies.

## 3 RESULTS

### 3.1 Behavioral categories

**Figure 2:**
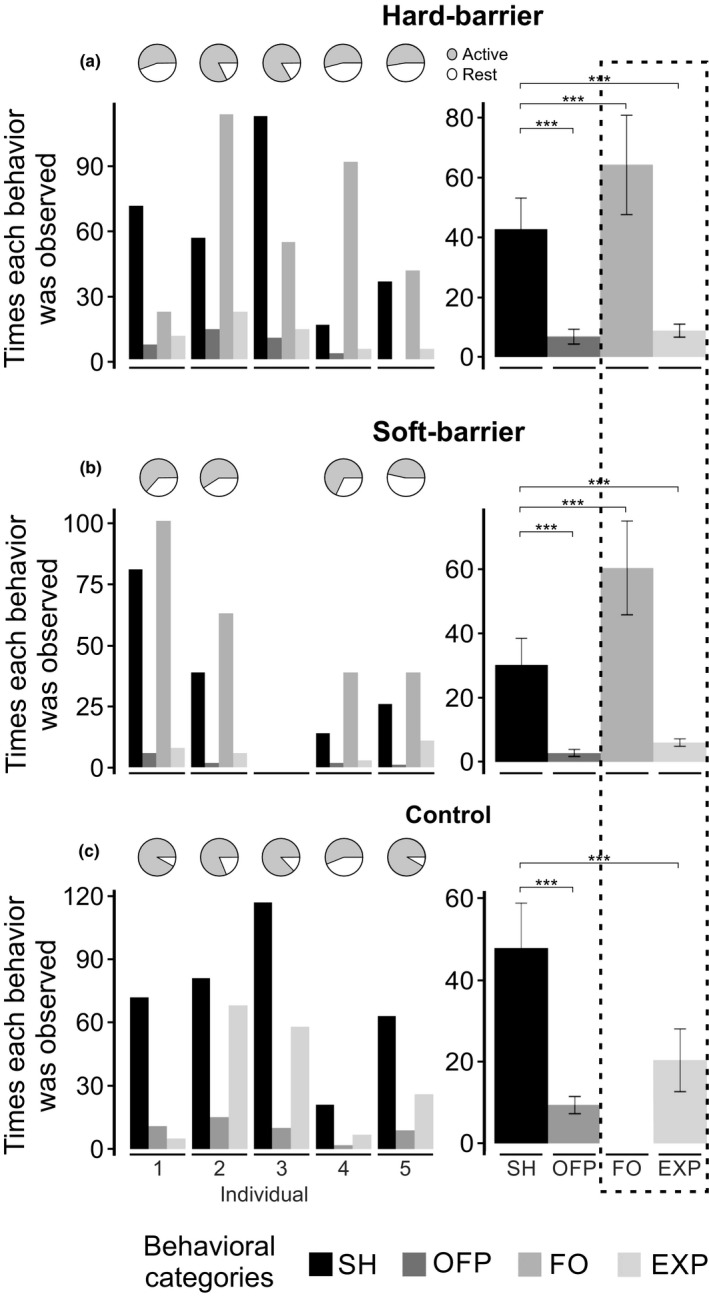
Number of times *Sorex unguiculatus* individuals displayed behaviors within the three following experimental conditions: (a) hard‐barrier, (b) soft‐barrier, and (c) control. The plots on the right represent the averaged time behaviors displayed among individuals, statistically compared within each condition. Facing obstacles and exploring (EXP) behaviors are indicated by a broken line square, as these behaviors have been considered to be the context in which the shrew's orientation signals are emitted. In control conditions, shrews could not exhibit FO since there were no obstacles present. The pie charts above the bar plots represent the average time in which individuals remained active during the experimental period (2 hr). The bar colors represent the behavioral categories registered under each experimental condition. *** *p < *0.001

The five individuals remained mostly active throughout the 2 hr of each experiment, except individual 3 in the soft‐barrier condition (Figure 2). The animals moved around the shelter and faced the acrylic plates and plastic plants more frequently than the approach to the food dish in the three experimental conditions. Shrews significantly showed the FO instead of other behavioral categories in soft‐ and hard‐barrier conditions (Figure 2a,b and Supporting Information Table S1). In the control condition, SH (movements around the shelter) was the most repeated behavior (Figure 2c and Supporting Information Table S1). Thus, when there were no obstacles in the experimental cage (i.e., control condition), shrews exhibited more frequently SH versus other behavioral categories (e.g., EXP).

### 3.2 Analysis of vocalizations

Tonal, click, and noisy vocalizations recorded for the five *S. unguiculatus* individuals were audible, also extended to the ultrasonic frequency range (5–44.7 kHz), and had short durations (3–40 ms). A total of 4,289 vocalizations were recorded, of which 1,513 under the control condition and 1,389 and 617 under the hard‐barrier or soft‐barrier experimental conditions, respectively.

#### 3.2.1 Effects of the experimental conditions on shrew's vocalizations during EXP behavior

**Figure 3:**
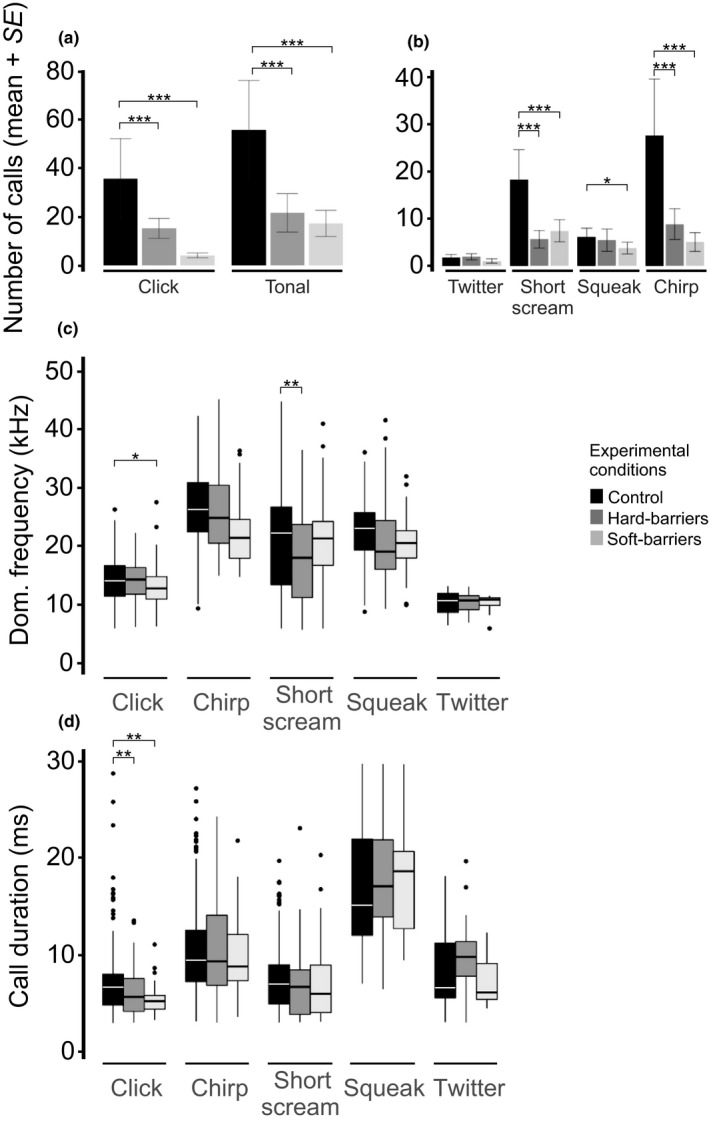
Call features from exploring behavior among experimental conditions. (a) Number of click and tonal calls emitted for this behavior. (b) Number of tonal type calls emitted during the experimental conditions. Mean dominant frequency (c) and call duration (d) of the click and tonal type calls. The bar colors represent the experimental conditions. * *p < *0.05, ** *p < *0.01, *** *p < *0.001

This analysis was performed when individuals underwent EXP behavior, in which animals moved around the cage without facing any particular item while moving fast and sniffing. This behavior was observed in all three conditions and has been previously proposed to be the context in which shrews’ orientation signals are emitted. In EXP, the number of tonal and click calls was more significant in the control condition than in the soft‐ and hard‐barrier conditions (Figure 3a and Supporting Information Table S2). Within the different types of tonal calls, short scream and chirp were more frequently emitted in the control condition than in the soft‐ and hard‐barrier conditions (Figure 3b and Supporting Information Table S2). Squeak calls were more frequently emitted in the control condition than in the soft‐barrier condition (Supporting Information Table S2).

The dominant frequencies of click calls were significantly lower in the soft‐barrier condition (*p* = 0.009) than in the control condition (Figure 3c and Supporting Information Table S3). Click durations were significantly lower in hard‐ (*p* = 0.001) and soft‐barrier (*p = *0.01) versus control conditions (Figure 3d). For the dominant frequencies of these tonal calls, the only significant differences were found for the short scream calls between the control and hard‐barrier conditions (*p = *0.005; Figure 3c and Supporting Information Table S3). The duration of these tonal calls was not significantly different amongst experimental conditions (Supporting Information Table S3).

#### 3.2.2 Effects of behavioral categories on vocalizations

##### Tonal calls

**Figure 4:**
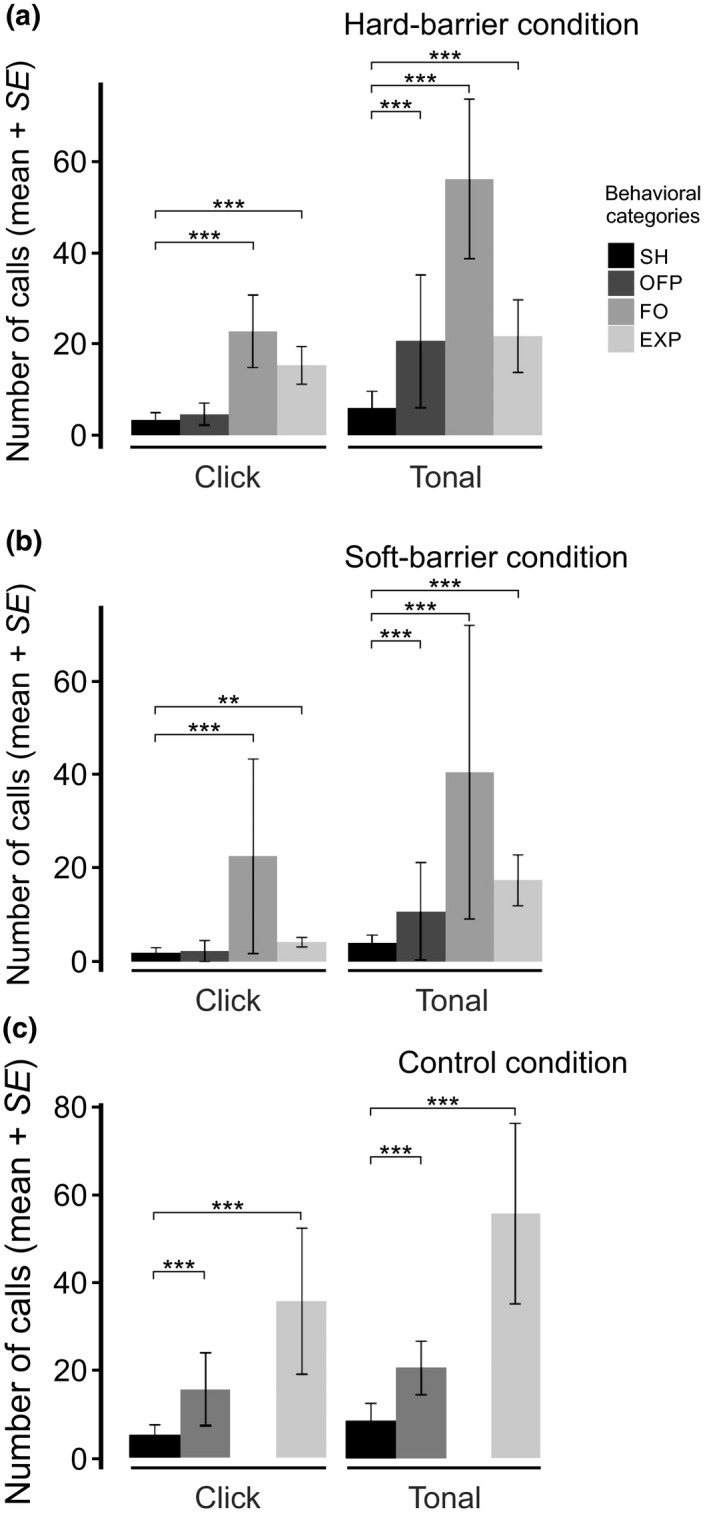
Average number of click and tonal vocalizations from five individuals of *Sorex unguiculatus* under three experimental conditions, (a) hard‐barrier, (b) soft‐barrier, and (c) control. The bar colors represent the behavioral categories registered under each experimental condition. ** *p < *0.01, *** *p < *0.001

**Figure 5:**
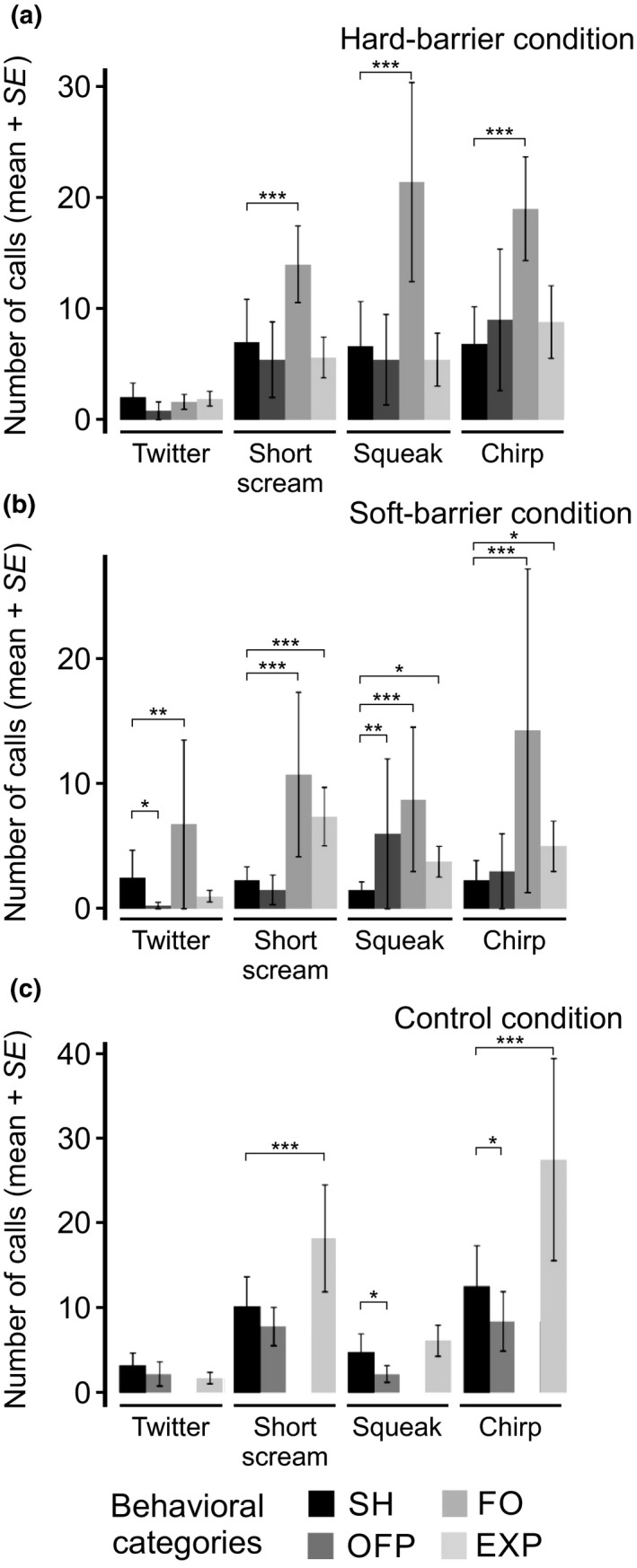
Average number of tonal type vocalizations from *Sorex unguiculatus* in three experimental conditions, (a) hard‐barrier, (b) soft‐barrier, and (c) control. The bar colors represent the behavioral categories registered under each experimental condition. * *p < *0.05, ** *p < *0.01, *** *p < *0.001

Analyzing within each experimental condition allowed to assess how the behavioral categories may have affected the number, duration, and dominant frequency of the vocalizations. The number of tonal calls significantly differed depending on the behavioral category under each experimental condition (Figure 4 and Supporting Information Table S4). The tonal calls were significantly the most frequently emitted when shrews encountered the acrylic plates or plants (FO) in the hard‐ and soft‐barrier conditions (Figure 4a,b, and Supporting Information Table S4), while these were the most frequent during EXP behavior in the control condition (Figure 4c and Supporting Information Table S4). When we analyzed the number of tonal type calls, short scream (*p = *0.0007), squeak (*p = *2.88 × 10^−09^), and chirp (*p = *2.42 × 10^−14^) were all emitted significantly during FO behavior in the hard‐barrier condition (Figure 5a). In the soft‐barrier condition, all call types (twitter, short scream, squeak, and chirp) were emitted most significantly during FO behavior (Figure 5b). Also, short scream, squeak, and chirp were emitted more significantly in EXP versus SH in this condition. In the control condition, in which FO could not be counted, short scream and chirp calls were more frequently emitted during EXP than SH (Figure 5c).

**Figure 6:**
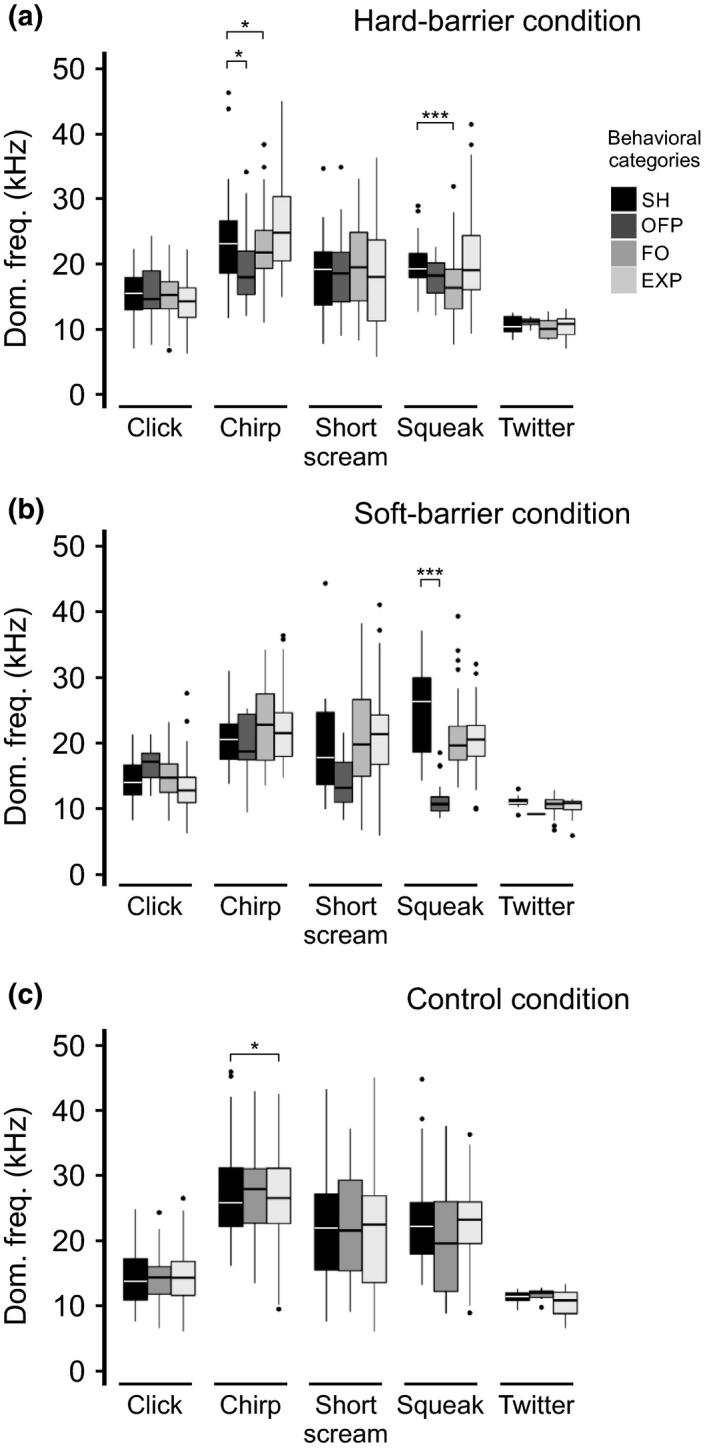
Mean dominant frequency of vocalizations recorded from five individuals of *Sorex unguiculatus* according to displayed behaviors, in the following three experimental conditions: (a) hard‐barrier, (b) soft‐barrier, and (c) control. The bar colors represent the call types registered in each experimental condition. * *p < *0.05, *** *p < *0.001

The dominant frequency of some tonal type calls was significantly affected by certain behaviors within experimental conditions. In the hard‐barrier condition, the dominant frequencies of chirp (*p = *0.047) and squeak (*p = *0.0002) calls were significantly lower during FO versus SH behavior (Figure 6a and Supporting Information Table S6). For chirp calls, the dominant frequency was also lower for OFP behavior (*p* = 0.027; Supporting Information Table S6). In the soft‐barrier condition, the dominant frequency of squeak calls was lower (*p = *1.15 × 10^−09^) for OFP in comparison with SH behavior (Figure 6b and Supporting Information Table S6). In the control condition, the dominant frequency of chirp was lower during EXP compared to SH (Figure 6c and Supporting Information Table S6).

**Figure 7:**
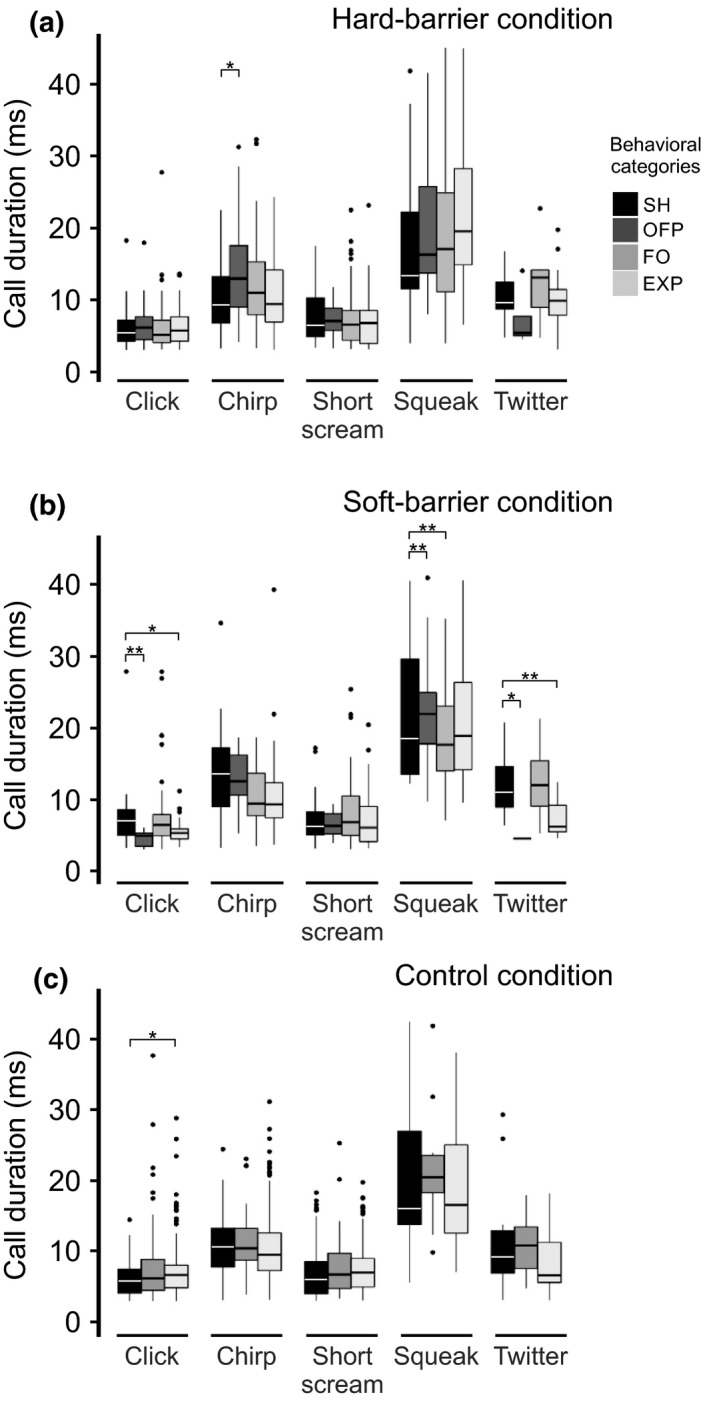
Duration of the vocalizations recorded from five individuals of *Sorex unguiculatus* according to displayed behaviors, in the following three experimental conditions: (a) hard‐barrier, (b) soft‐barrier, and (c) control. The bar colors represent call types registered in each experimental condition. * *p < *0.05, ** *p < *0.01

The call durations in FO and EXP were significantly lower than that of SH for squeak and twitter in the soft‐barrier condition (Figure 7b and Supporting Information Table S7). In the hard‐barrier condition, the duration of chirp calls was significantly higher in OFP (*p = *0.031) compared to SH (Figure 7a and Supporting Information Table S7). There were no significant differences in the call duration of any types of tonal calls among the behavioral categories (Figure 7c and Supporting Information Table S7).

##### Click calls

As for tonal calls, the click calls were most frequently and significantly emitted when shrews faced the acrylic plates or plants (FO) in the hard‐ and soft‐barrier conditions, respectively (Figure 4a,b, and Supporting Information Table S4), while these were the most frequent during EXP behavior in the control condition (Figure 4c and Supporting Information Table S4).

The dominant frequency of click calls was not affected by the behaviors in any of the experimental conditions (Figure 6, Supporting Information Table S6). In the soft‐barrier condition, the duration of the click calls was lower during OFP (*p = *0.004) and higher during EXP (*p* = 0.031) in comparison with SH (Figure 7b). In the control condition, the call duration was higher during EXP (*p* = 0.023) versus SH behavior (Figure 7c and Supporting Information Table S7).

## 4 DISCUSSION

Echolocation ability in Eulipotyphla has been argued for several years, and their vocal behavior has been tested with several experiments (Buchler, 1976; Forsman & Malmquist, 1988; Thomas & Jalili, 2004). Particularly for shrews, click vocalizations were recorded using bat detectors while examined the environment. These investigators referred to echolocation mainly based on the high‐frequency emissions found in those shrew species and the context of probing an environment. Basing the echolocation criterion, exclusively on the use of high‐frequency calls might be complicated since other species vocalize via ultrasound for communication but not orientation purposes (Ramsier et al., 2012; Sirotin, Costa, & Laplagne, 2014; Wöhr & Schwarting, 2007). However, a more recent study proposed a novel terminology for this vocal behavior in shrews for the same type of behavior, echo‐based orientation (Siemers et al., 2009). Our results support this hypothesis since shrews vocalized more frequently when they probed their environments, that is, during FO behavior in soft‐ and hard‐barrier conditions, and exploring (EXP) behavior in control conditions (Figure 4).

Our results showed that among experimental conditions, shrews vocalized more in the absence of obstacles (Figure 3a,b). These results were not consistent with a previous study by Siemers et al. (2009) showing that the least number of calls were observed when shrews faced a noncomplex environment. One of the reasons for the different results might be due to different methods for recording vocalizations in the both studies. We automatically detected each one of the emitted calls and furtherly characterized them making a classification of it according to each type. Also, in the previous article, authors focused apparently in one type of call, possibly twitter calls according to their description. As our results show here, shrews can emit a diversity of calls depending on their behavior and environment (Figure 3). Shrews vocalized less frequently in the soft‐ or hard‐barrier conditions since they tend to remain around the acrylic plate or crawl amongst plastic plants, and they emitted calls more frequently in the control condition since they continued to search through their environment. Recent studies strongly support that somatosensory information gathered through the Etruscan shrew's (*Suncus etruscus*) whiskers appear to be relevant in allowing them to detect obstacles and forage (Anjum, Turni, Mulder, Burg, & Brecht, 2006; Catania, 1999; Catania & Henry, 2006; Munz, Brecht, & Wolfe, 2010). Vocalizations are unlikely to be emitted during the shrew's final phase of prey capture (Catania, Hare, & Campbell, 2008; Catania 2012). In the experiments presented here, shrews might be using these emitted vocalizations for rough scanning of the surrounding environments when encountering obstacles, and somatosensory information obtained through sniffing subsequently allowed them to obtain more detailed information.

Siemers et al. (2009) tested whether tonal calls could convey echoes from obstacles commonly present in the shrew's environment. Even when not analyzed the echoes from the obstacles in our experimental conditions, the results obtained from the shrew vocalizations within each of the experimental conditions strongly suggest that these animals partially rely on acoustic information when examining their environment. Shrews modified some acoustic features, such as dominant frequency, duration, and used different calls (i.e., as shown by the different frequency patterns observed in the spectrograms). The present results indicated that all the call types—except twitter—were emitted most frequently during FO behavior. Twitter was most frequently emitted during FO only in the soft‐barrier condition. However, for EXP behavior, short scream and chirp were more frequently emitted in the control condition (Figure 3). This result suggests that the shrew modifies the type of calls (i.e., calls differing in their frequency patterns) during different behaviors and for the varying obstacles that they encounter. When we focused on FO behavior, we found several significant differences in the dominant frequencies and call durations among different behavioral categories. The dominant frequency of squeak was significantly lower in the hard‐barrier condition (Figure 6a), and its duration was significantly lower in the soft‐barrier condition amongst all behavioral categories (Figure 7b). In addition, when we compared the number of calls and acoustic features during FO behavior between soft‐ and hard‐barrier conditions, the number of squeaks was significantly higher, and the dominant frequency was lower in the soft‐barrier versus hard‐barrier conditions (Supporting Information Figure S3 and Supporting Information Tables S8 and S9). These data indicate that shrews also modify the dominant frequency, especially of squeak calls, between different types of barriers (Supporting Information Figure S3c). In addition, obstacles may impose a change in the number of shrew's emitted twitter calls (Supporting Information Figure S3b). Previous studies have reported shrews’ twitter calls (Schneiderová, 2014; Siemers et al., 2009; Zsebők et al., 2015) in more detail than squeak calls (Schneiderová, 2014) and showed that squeaks are emitted more frequently when shrews move around housing cages. This result could also suggest that shrews switch among different types of calls depending on the behavior developed (Supporting Information Figure S2). However, the number of each type of vocalization differed depending on the behavior displayed by shrews, as mentioned previously. This particular emission of vocalizations within certain behaviors could be further investigated by designing particular environments in order to enhance these vocalizations (i.e., by placing various obstacles or more novel environments).

Echolocating species show some unique vocal emission patterns (Schnitzler, Moss, & Denzinger, 2003; Stimpert, Wiley, Au, Johnson, & Arsenault, 2007). An obvious approaching sequence with several vocalizations emitted at short inter‐pulse intervals can be observed in vocalization waveforms or spectrograms when such animals approach obstacles, indicating feedback that relies on the perceived acoustic information (Neuweiler, 1984). In this study, we did not observe vocalizations emitted with short inter‐pulse intervals (Supporting Information Figure S2). A great number of echolocating species use a particular call with a unique frequency pattern in their echolocation calls. These calls are consistently repeated by these animals and can be used as an identification tool to recognize species (Fenton & Bell, 1981; Russo & Jones, 2002). For shrews, some vocalizations such as twitter have been proposed to allow the identification of several European shrew species (Zsebők et al., 2015). However, most of the studies describing the vocal repertoire of shrews (Schneiderová, 2014; Volodin et al., 2015) have not still identified a distinct, highly repeated pattern used within a particular behavior such as facing obstacles and exploring.

Echo‐based orientation appears to be a simpler system in comparison with echolocation, in which sound might not constitute the primary sense by which shrews gather information from environments. In echolocation, specific calls are used to extract information from the environment, and these emitted calls are adjusted when individuals approach obstacles. In echo‐based orientation, changing the calling rate seems to convey more information to shrews in contrast to call parameter adjustments like echolocation. In our study, we tested whether shrews could modify the acoustic features of their vocalizations within three experimental conditions. The results showed that shrews increased their call rate when probing the environment, that is, when facing obstacles or walking around the cage. Shrews emitted clicks and several different types of tonal calls when encountering obstacles or exploring, and they modified the usage of different types of calls for varying behavior.

Furthermore, shrews adjusted the dominant frequency and duration of squeak calls for different types of obstacles, that is, plants and acrylic barriers. These results indicate that the shrew *S. unguiculatus* uses a simple echo‐based orientation system to obtain information from their surrounding environment. Further studies are needed in order to describe in more detail the mechanisms of the shrew's orientation system.

## AUTHOR CONTRIBUTIONS

L.S., L.E., and M. K. conceived the study. L. S. and M. K designed the experiment. L. S. conducted experiments. L.S., A. K., S. O. collected the samples. L. S. conducted and analyzed data. L.S., S. M. and M. K wrote the manuscript. All authors read and approved the final manuscript.

## Data Availability

Data available from the Dryad Digital Repository: https://doi.org/10.5061/dryad.pt70k51.
