# Supplementary material for: Acoustic emissions of Sorex unguiculatus (Mammalia: Soricidae): Assessing the echo‐based orientation hypothesis
Source: Ecol Evol. 2019 Feb 15;9(5):2629–39. doi: 10.1002/ece3.4930 (PMC6405488; doi:10.1002/ece3.4930)
Supplement: Supplementary file 1 [file ECE3-9-2629-s001.docx]

**FIGURE S1** Experimental conditions for conducting behavioral experiments on *Sorex unguiculatus*. (a) Hard-barrier, (b) soft-barrier, and (c) control. X represents the distance among the set obstacles, which was the same for both (a) and (b) conditions.

**FIGURE S2** Waveforms and spectrograms of sound files recorded from one individual of *Sorex unguiculatus* while facing obstacle behavior (FO) and standing on the food plate (OFP) (a–b) in the hard-barrier and (c**–**d) soft-barrier conditions.

**FIGURE S3** Call features during facing obstacles behavior (FO) between two different types of obstacles (plant and acrylic plates). (a) The number of click and tonal calls emitted between experimental conditions. (b) The number of tonal type calls emitted in the experimental conditions. Mean dominant frequency (c) and duration (d) of click and tonal type calls. The bar colors represent the experimental conditions. * *P* < 0.05, ** *P* < 0.01, *** *P* < 0.001.
