# Supplementary material for: Acoustic emissions of Sorex unguiculatus (Mammalia: Soricidae): Assessing the echo‐based orientation hypothesis
Source: Ecol Evol. 2019 Feb 15;9(5):2629–39. doi: 10.1002/ece3.4930 (PMC6405488; doi:10.1002/ece3.4930)
Supplement: Supplementary file 2 [file ECE3-9-2629-s002.docx]

**Table S1.** Results of the generalized linear mixed model for comparing the number of different behavioral categories exhibited by the individuals in each experimental condition. SH behavior was used as a reference in the analysis. The number of animals is included in the model as a random effect.

|  | Estimate | Std. Error | Z value | P value |
| --- | --- | --- | --- | --- |
| Hard-barrier condition |  |  |  |  |
| On the food plate | -1.837 | 0.178 | -10.324 | <2x10^-16^ |
| Facing obstacles | 0.0408 | 0.074 | 5.528 | 3.25 x10^-08^ |
| Exploring | -1.579 | 0.117 | -13.509 | <2x10^-16^ |
|  |  |  |  |  |
| Soft-barrier condition |  |  |  |  |
| On the food plate | -2.397 | 0.308 | -7.791 | 6.64x10^-15^ |
| Facing obstacles | 0.693 | 0.091 | 7.637 | 2.22x10^-14^ |
| Exploring | -1.618 | 0.158 | -10.256 | <2x10^-16^ |
|  |  |  |  |  |
| Control condition |  |  |  |  |
| On the food plate | -1.63 | 0.152 | -10.69 | <2x10^-16^ |
| Exploring | -0.856 | 0.083 | -10.26 | <2x10^-16^ |

**Table S2.** Results for the generalized linear mixed model for the effects of the three experimental conditions on the number of vocalizations within the EXP behavioral category. The control condition was used as a reference in the analysis. The number of animals was included in the model as a random effect.

| Click | Estimate | Std. Error | Z value | P value |
| --- | --- | --- | --- | --- |
| Hard-barrier | -0.847 | 0.096 | -8.787 | <2x10^-16^ |
| Soft-barrier | -1.693 | 0.186 | -9.074 | <2x10^-16^ |

| Tonal | Estimate | Std. Error | Z value | P value |
| --- | --- | --- | --- | --- |
| Hard-barrier | -0.944 | 0.079 | -11.821 | <2x10^-16^ |
| Soft-barrier | -0.913 | 0.099 | -9.252 | <2x10^-16^ |

| Chirp | Estimate | Std. Error | z value | P value |
| --- | --- | --- | --- | --- |
| Hard-barrier | -1.139 | 0.122 | -9.33 | <2x10^-16^ |
| Soft-barrier | -1.31 | 0.175 | -7.473 | 7.86x10^-14^ |

| Squeak | Estimate | Std. Error | z value | P value |
| --- | --- | --- | --- | --- |
| Hard-barrier | -0.122 | 0.186 | -0.657 | 0.511 |
| Soft-barrier | -0.449 | 0.227 | -1.984 | 0.047 |

| Short scream | Estimate | Std. Error | z value | P value |
| --- | --- | --- | --- | --- |
| Hard-barrier | -1.179 | 0.152 | -7.742 | 9.79x10^-15^ |
| Soft-barrier | -0.675 | 0.157 | -4.301 | 1.7x10^-05^ |

| Twitter | Estimate | Std. Error | z value | P value |
| --- | --- | --- | --- | --- |
| Hard-barrier | 0.111 | 0.327 | 0.34 | 0.734 |
| Soft-barrier | -0.418 | 0.432 | -0.968 | 0.333 |

**Table S3.** Results of the generalized linear mixed model for the effects of the three experimental conditions on call duration and dominant frequency of click, twitter, short scream, squeak and chirp calls within the EXP behavioral category. The control condition was used as a reference in the analysis. The number of animals was included in the model as a random effect.

| Click | Estimate | Std. Error | t value | P value |
| --- | --- | --- | --- | --- |
| Dominant Frequency |  |  |  |  |
| Hard-barrier | -3.427 | 1.149 | -0.03 | 0.976 |
| Soft-barrier | -5.757 | 2.204 | -2.612 | 0.009 |
|  |  |  |  |  |
| Call Duration |  |  |  |  |
| Hard-barrier | -0.051 | 0.016 | -3.2 | 0.001 |
| Soft-barrier | -0.079 | 0.031 | -2.588 | 0.01 |

| Chirp | Estimate | Std. Error | t value | P value |
| --- | --- | --- | --- | --- |
| Dominant Frequency |  |  |  |  |
| Hard-barrier | 0.008 | 0.013 | 0.587 | 0.557 |
| Soft-barrier | -0.031 | 0.019 | -1.662 | 0.097 |
|  |  |  |  |  |
| Call Duration |  |  |  |  |
| Hard-barrier | -0.004 | 0.025 | -0.173 | 0.863 |
| Soft-barrier | 0.009 | 0.035 | 0.279 | 0.781 |

| Short scream | Estimate | Std. Error | t value | P value |
| --- | --- | --- | --- | --- |
| Dominant Frequency |  |  |  |  |
| Hard-barrier | -0.087 | 0.031 | -2.831 | 0.005 |
| Soft-barrier | -0.038 | 0.03 | -1.256 | 0.21 |
|  |  |  |  |  |
| Call Duration |  |  |  |  |
| Hard-barrier | -0.041 | 0.03 | -1.353 | 0.177 |
| Soft-barrier | -0.043 | 0.029 | -1.449 | 0.148 |

| Squeak | Estimate | Std. Error | t value | P value |
| --- | --- | --- | --- | --- |
| Dominant Frequency |  |  |  |  |
| Hard-barrier | -0.045 | 0.025 | -1.816 | 0.072 |
| Soft-barrier | -0.044 | 0.029 | -1.493 | 0.137 |
|  |  |  |  |  |
| Call Duration |  |  |  |  |
| Hard-barrier | 0.054 | 0.044 | 1.226 | 0.229 |
| Soft-barrier | 0.073 | 0.049 | 1.472 | 0.144 |

| Twitter | Estimate | Std. Error | t value | P value |
| --- | --- | --- | --- | --- |
| Dominant Frequency |  |  |  |  |
| Hard-barrier | 0.012 | 0.029 | 0.404 | 0.688 |
| Soft-barrier | -0.003 | 0.038 | -0.077 | 0.94 |
|  |  |  |  |  |
| Call Duration |  |  |  |  |
| Hard-barrier | 0.057 | 0.068 | 0.835 | 0.408 |
| Soft-barrier | -0.056 | 0.088 | -0.638 | 0.527 |

**Table S4.** Results of the generalized linear mixed model for different behavioral categories for the number of click and tonal vocalizations within each experimental condition. SH behavioral category was used as a reference in the analysis. The number of individuals was included in the model as a random effect.

| Click | Estimate | Std. Error | Z value | P value |
| --- | --- | --- | --- | --- |
| Hard-barrier condition |  |  |  |  |
| On the food plate | 0.347 | 0.242 | 1.437 | 0.151 |
| Facing obstacles | 1.948 | 0.155 | 12.576 | <2x10^-16^ |
| Exploring | 1.549 | 0.148 | 10.498 | <2x10^-16^ |
|  |  |  |  |  |
| Soft-barrier condition |  |  |  |  |
| On the food plate | 0.216 | 0.379 | 0.57 | 0.569 |
| Facing obstacles | 2.519 | 0.212 | 11.861 | <2x10^-16^ |
| Exploring | 0.822 | 0.253 | 3.249 | 0.001 |
|  |  |  |  |  |
| Control condition |  |  |  |  |
| On the food plate | 1.108 | 0.149 | 7.4 | 1.36x10^-13^ |
| Exploring | 1.936 | 0.112 | 17.35 | <2x10^-16^ |

| Tonal | Estimate | Std. Error | Z value | P value |
| --- | --- | --- | --- | --- |
| Hard-barrier condition |  |  |  |  |
| On the food plate | 1.225 | 0.134 | 9.154 | <2x10^-16^ |
| Facing obstacles | 2.229 | 0.109 | 20.532 | <2x10^-16^ |
| Exploring | 1.277 | 0.113 | 11.276 | <2x10^-16^ |
|  |  |  |  |  |
| Soft-barrier condition |  |  |  |  |
| On the food plate | 1.02 | 0.198 | 5.154 | 2.56x10^-07^ |
| Facing obstacles | 2.347 | 0.149 | 15.751 | <2x10^-16^ |
| Exploring | 1.5 | 0.152 | 9.848 | <2x10^-16^ |
|  |  |  |  |  |
| Control condition |  |  |  |  |
| On the food plate | 0.874 | 0.124 | 7.021 | 2.2x10^-12^ |
| Exploring | 1.87 | 0.087 | 21.473 | <2x10^-16^ |

**Table S5**. Results of the generalized linear mixed model for the effect of different behavioral categories on the number of twitter, short scream, squeak, and chirp calls in each experimental condition. SH behavioral category was used as a reference in the analysis. The number of individuals was included in the model as a random effect.

| Twitter | Estimate | Std. Error | Z value | P value |
| --- | --- | --- | --- | --- |
| Hard-barrier condition |  |  |  |  |
| On the food plate | -0.916 | 0.578 | -1.583 | 0.114 |
| Facing obstacles | -0.223 | 0.464 | -0.481 | 0.631 |
| Exploring | -0.051 | 0.382 | -0.134 | 0.893 |
|  |  |  |  |  |
| Soft-barrier condition |  |  |  |  |
| On the food plate | -2.303 | 1.049 | -2.195 | 0.028 |
| Facing obstacles | 0.993 | 0.37 | 2.683 | 0.007 |
| Exploring | -0.916 | 0.474 | -1.932 | 0.053 |
|  |  |  |  |  |
| Control condition |  |  |  |  |
| On the food plate | -0.375 | 0.385 | -0.974 | 0.329 |
| Exploring | -0.633 | 0.342 | -1.85 | 0.064 |

| Short scream | Estimate | Std. Error | Z value | P value |
| --- | --- | --- | --- | --- |
| Hard-barrier condition |  |  |  |  |
| On the food plate | -0.259 | 0.255 | -1.019 | 0.308 |
| Facing obstacles | 0.693 | 0.206 | 3.367 | 0.0007 |
| Exploring | -0.223 | 0.214 | -1.042 | 0.298 |
|  |  |  |  |  |
| Soft-barrier condition |  |  |  |  |
| On the food plate | -0.406 | 0.523 | -0.775 | 0.438 |
| Facing obstacles | 1.564 | 0.364 | 4.296 | 1.74x10^-16^ |
| Exploring | 1.187 | 0.355 | 3.341 | 0.0008 |
|  |  |  |  |  |
| Control condition |  |  |  |  |
| On the food plate | -0.268 | 0.212 | -1.266 | 0.205 |
| Exploring | 0.579 | 0.158 | 3.67 | 0.0002 |

| Squeak | Estimate | Std. Error | Z value | P value |
| --- | --- | --- | --- | --- |
| Hard-barrier condition |  |  |  |  |
| On the food plate | -0.201 | 0.258 | -0.777 | 0.437 |
| Facing obstacles | 1.176 | 0.198 | 5.938 | 2.88x10^-09^ |
| Exploring | -0.201 | 0.219 | -0.913 | 0.361 |
|  |  |  |  |  |
| Soft-barrier condition |  |  |  |  |
| On the food plate | 1.386 | 0.452 | 3.064 | 0.002 |
| Facing obstacles | 1.764 | 0.438 | 4.027 | 5.65x10^-05^ |
| Exploring | 0.916 | 0.443 | 2.067 | 0.039 |
|  |  |  |  |  |
| Control condition |  |  |  |  |
| On the food plate | -0.78 | 0.361 | -2.163 | 0.031 |
| Exploring | 0.239 | 0.238 | 1.004 | 0.315 |

| Chirp | Estimate | Std. Error | Z value | P value |
| --- | --- | --- | --- | --- |
| Hard-barrier condition |  |  |  |  |
| On the food plate | 0.28 | 0.226 | 1.239 | 0.215 |
| Facing obstacles | 1.027 | 0.199 | 5.164 | 2.42x10^-14^ |
| Exploring | 0.258 | 0.201 | 1.282 | 0.2 |
|  |  |  |  |  |
| Soft-barrier condition |  |  |  |  |
| On the food plate | 0.288 | 0.438 | 0.657 | 0.511 |
| Facing obstacles | 1.846 | 0.356 | 5.184 | 2.17x10^-07^ |
| Exploring | 0.798 | 0.366 | 2.18 | 0.029 |
|  |  |  |  |  |
| Control condition |  |  |  |  |
| On the food plate | -0.406 | 0.199 | -2.042 | 0.041 |
| Exploring | 0.781 | 0.139 | 5.605 | 2.09x10^-08^ |

**Table S6.** Results of the generalized linear model for the effects of different behavioral categories on the dominant frequency of click and tonal type calls within each experimental condition. SH behavioral category was used as a reference in the analysis. The number of animals was included in the model as a random effect.

| Click vocalizations | Estimate | Std. Error | t value | P value |
| --- | --- | --- | --- | --- |
| Hard-barrier condition |  |  |  |  |
| On the food plate | 3.608 | 2.797 | 0.129 | 0.897 |
| Facing obstacles | 6.197 | 1.772 | 0.035 | 0.972 |
| Exploring | -3.125 | 1.693 | -1.846 | 0.066 |
|  |  |  |  |  |
| Soft-barrier condition |  |  |  |  |
| On the food plate | 0.071 | 0.041 | 1.731 | 0.085 |
| Facing obstacles | 0.018 | 0.023 | 0.789 | 0.432 |
| Exploring | -0.041 | 0.029 | -1.408 | 0.167 |
|  |  |  |  |  |
| Control condition |  |  |  |  |
| On the food plate | 0.015 | 0.018 | 0.818 | 0.414 |
| Exploring | 0.016 | 0.014 | 1.162 | 0.246 |

| Chirp vocalizations | Estimate | Std. Error | t value | P value |
| --- | --- | --- | --- | --- |
| Hard-barrier condition |  |  |  |  |
| On the food plate | -0.051 | 0.023 | -2.22 | 0.027 |
| Facing obstacles | -0.042 | 0.021 | -1.999 | 0.047 |
| Exploring | 0.015 | 0.021 | 0.702 | 0.483 |
|  |  |  |  |  |
| Soft-barrier condition |  |  |  |  |
| On the food plate | -0.038 | 0.042 | -0.917 | 0.36 |
| Facing obstacles | 0.045 | 0.03 | 1.466 | 0.145 |
| Exploring | 0.011 | 0.032 | 0.348 | 0.728 |
|  |  |  |  |  |
| Control condition |  |  |  |  |
| On the food plate | -0.02 | 0.019 | -1.009 | 0.314 |
| Exploring | -0.032 | 0.014 | -2.323 | 0.021 |

| Short scream vocalizations | Estimate | Std. Error | t value | P value |
| --- | --- | --- | --- | --- |
| Hard-barrier condition |  |  |  |  |
| On the food plate | 0.034 | 0.042 | 0.814 | 0.417 |
| Facing obstacles | 0.06 | 0.037 | 1.634 | 0.104 |
| Exploring | 0.011 | 0.038 | 0.288 | 0.773 |
|  |  |  |  |  |
| Soft-barrier condition |  |  |  |  |
| On the food plate | -0.128 | 0.078 | -1.637 | 0.104 |
| Facing obstacles | 0.029 | 0.046 | 0.637 | 0.525 |
| Exploring | 0.034 | 0.044 | 0.778 | 0.438 |
|  |  |  |  |  |
| Control condition |  |  |  |  |
| On the food plate | 2.16 | 4.102 | 0.528 | 0.598 |
| Exploring | 7.05 | 3.014 | 0.234 | 0.815 |

| Squeak vocalizations | Estimate | Std. Error | t value | P value |
| --- | --- | --- | --- | --- |
| Hard-barrier condition |  |  |  |  |
| On the food plate | -0.041 | 0.03 | -1.335 | 0.183 |
| Facing obstacles | -0.087 | 0.023 | -3.805 | 0.0002 |
| Exploring | 0.003 | 0.026 | 0.103 | 0.918 |
|  |  |  |  |  |
| Soft-barrier condition |  |  |  |  |
| On the food plate | -0.267 | 0.039 | -6.694 | 1.15x10^-09^ |
| Facing obstacles | -0.027 | 0.034 | -0.788 | 0.432 |
| Exploring | -0.061 | 0.033 | -1.87 | 0.064 |
|  |  |  |  |  |
| Control condition |  |  |  |  |
| On the food plate | -0.082 | 0.049 | -1.684 | 0.095 |
| Exploring | -0.009 | 0.031 | -0.305 | 0.761 |

| Twitter vocalizations | Estimate | Std. Error | t value | P value |
| --- | --- | --- | --- | --- |
| Hard-barrier condition |  |  |  |  |
| On the food plate | 0.018 | 0.041 | 0.44 | 0.662 |
| Facing obstacles | -0.014 | 0.033 | -0.42 | 0.677 |
| Exploring | -0.014 | 0.028 | -0.498 | 0.624 |
|  |  |  |  |  |
| Soft-barrier condition |  |  |  |  |
| On the food plate | -0.086 | 0.067 | -1.285 | 0.206 |
| Facing obstacles | -0.031 | 0.022 | -1.384 | 0.173 |
| Exploring | -0.041 | 0.029 | -1.399 | 0.169 |
|  |  |  |  |  |
| Control condition |  |  |  |  |
| On the food plate | 0.011 | 0.026 | 0.428 | 0.671 |
| Exploring | -0.033 | 0.024 | -1.371 | 0.178 |

**Table S7.** Results of the generalized linear model for the effects of different behavioral categories on the call duration for click and tonal type calls within each experimental condition. The SH behavioral category was used as a reference in the analysis. The number of animals was included in the model as a random effect.

| Click vocalizations | Estimate | Std. Error | t value | P value |
| --- | --- | --- | --- | --- |
| Hard-barrier condition |  |  |  |  |
| On the food plate | 4.954 | 3.97 | 1.248 | 0.213 |
| Facing obstacles | 4.122 | 2.538 | 0.162 | 0.871 |
| Exploring | 2.157 | 2.419 | 0.892 | 0.373 |
|  |  |  |  |  |
| Soft-barrier condition |  |  |  |  |
| On the food plate | -0.197 | 0.067 | -2.949 | 0.004 |
| Facing obstacles | -0.026 | 0.038 | -0.692 | 0.489 |
| Exploring | -0.108 | 0.049 | -2.181 | 0.031 |
|  |  |  |  |  |
| Control condition |  |  |  |  |
| On the food plate | 0.043 | 0.028 | 1.551 | 0.122 |
| Exploring | 0.048 | 0.021 | 2.292 | 0.023 |

| Chirp vocalizations | Estimate | Std. Error | t value | P value |
| --- | --- | --- | --- | --- |
| Hard-barrier condition |  |  |  |  |
| On the food plate | 0.104 | 0.048 | 2.166 | 0.031 |
| Facing obstacles | 0.053 | 0.043 | 1.238 | 0.217 |
| Exploring | -0.005 | 0.043 | -0.115 | 0.908 |
|  |  |  |  |  |
| Soft-barrier condition |  |  |  |  |
| On the food plate | 0.0005 | 0.074 | 0.006 | 0.995 |
| Facing obstacles | -0.095 | 0.054 | -1.784 | 0.081 |
| Exploring | -0.094 | 0.056 | -1.676 | 0.099 |
|  |  |  |  |  |
| Control condition |  |  |  |  |
| On the food plate | 0.002 | 0.039 | 0.042 | 0.966 |
| Exploring | -0.038 | 0.027 | -1.413 | 0.159 |

| Short scream vocalizations | Estimate | Std. Error | t value | P value |
| --- | --- | --- | --- | --- |
| Hard-barrier condition |  |  |  |  |
| On the food plate | -0.007 | 0.051 | -0.147 | 0.883 |
| Facing obstacles | -0.022 | 0.041 | -0.534 | 0.594 |
| Exploring | -0.05 | 0.043 | -1.171 | 0.243 |
|  |  |  |  |  |
| Soft-barrier condition |  |  |  |  |
| On the food plate | -0.04 | 0.104 | -0.388 | 0.699 |
| Facing obstacles | 0.029 | 0.061 | 0.482 | 0.631 |
| Exploring | -0.044 | 0.057 | -0.767 | 0.445 |
|  |  |  |  |  |
| Control condition |  |  |  |  |
| On the food plate | 0.037 | 0.042 | 0.889 | 0.375 |
| Exploring | 0.038 | 0.03 | 1.245 | 0.215 |

| Squeak vocalizations | Estimate | Std. Error | t value | P value |
| --- | --- | --- | --- | --- |
| Hard-barrier condition |  |  |  |  |
| On the food plate | 2.587 | 6.254 | 0.041 | 0.967 |
| Facing obstacles | 2.071 | 4.704 | 0.044 | 0.965 |
| Exploring | 7.111 | 5.271 | 1.349 | 0.186 |
|  |  |  |  |  |
| Soft-barrier condition |  |  |  |  |
| On the food plate | -0.221 | 0.073 | -3.025 | 0.003 |
| Facing obstacles | -0.257 | 0.062 | -4.134 | 7.25x10^-05^ |
| Exploring | -0.095 | 0.059 | -1.599 | 0.113 |
|  |  |  |  |  |
| Control condition |  |  |  |  |
| On the food plate | 0.033 | 0.073 | 0.456 | 0.649 |
| Exploring | -0.021 | 0.046 | -0.46 | 0.646 |

| Twitter vocalizations | Estimate | Std. Error | t value | P value |
| --- | --- | --- | --- | --- |
| Hard-barrier condition |  |  |  |  |
| On the food plate | -0.178 | 0.121 | -1.466 | 0.151 |
| Facing obstacles | 0.041 | 0.097 | 0.427 | 0.672 |
| Exploring | -0.041 | 0.079 | -0.515 | 0.616 |
|  |  |  |  |  |
| Soft-barrier condition |  |  |  |  |
| On the food plate | -0.399 | 0.165 | -2.416 | 0.019 |
| Facing obstacles | 0.004 | 0.054 | 0.068 | 0.946 |
| Exploring | -0.215 | 0.072 | -3.012 | 0.004 |
|  |  |  |  |  |
| Control condition |  |  |  |  |
| On the food plate | 0.029 | 0.09 | 0.317 | 0.753 |
| Exploring | -0.066 | 0.08 | -0.818 | 0.418 |

**Table S8.** Results of the generalized linear model for the effects of two different type of obstacles on the number of click, chirp, short scream, squeak, and twitter calls during FO behavior. The hard-barrier condition was used as a reference in the analysis. The number of animals was included in the model as a random effect.

| Click | Estimate | Std. Error | Z value | P value |
| --- | --- | --- | --- | --- |
| Soft-barrier condition | 0.159 | 0.154 | 1.036 | 0.3 |

| Tonal | Estimate | Std. Error | Z value | P value |
| --- | --- | --- | --- | --- |
| Soft-barrier condition | -0.172 | 0.106 | -1.621 | 0.105 |

| Chirp | Estimate | Std. Error | Z value | P value |
| --- | --- | --- | --- | --- |
| Soft-barrier condition | -0.149 | 0.179 | -0.837 | 0.403 |

| Short scream | Estimate | Std. Error | Z value | P value |
| --- | --- | --- | --- | --- |
| Soft-barrier condition | -0.228 | 0.199 | -1.143 | 0.253 |

| Squeak | Estimate | Std. Error | Z value | P value |
| --- | --- | --- | --- | --- |
| Soft-barrier condition | -0.686 | 0.205 | -3.339 | 0.0008 |

| Twitter | Estimate | Std. Error | Z value | P value |
| --- | --- | --- | --- | --- |
| Soft-barrier condition | 1.614 | 0.469 | 3.438 | 0.0006 |

**Table S9.** The results of the generalized linear model for the effects of two different types of obstacles on the dominant frequency and duration of click, chirp, short scream, squeak, and twitter calls during FO behavior. The hard-barrier condition was used as a reference in the analysis. The number of animals was included in the model as a random effect.

| Click | Estimate | Std. Error | t value | P value |
| --- | --- | --- | --- | --- |
| Dominant Frequency |  |  |  |  |
| Soft-barrier condition | -0.029 | 0.02 | -1.474 | 0.147 |
|  |  |  |  |  |
| Call Duration |  |  |  |  |
| Soft-barrier condition | 0.079 | 0.025 | 3.165 | 0.002 |

| Chirp | Estimate | Std. Error | t value | P value |
| --- | --- | --- | --- | --- |
| Dominant Frequency |  |  |  |  |
| Soft-barrier condition | 0.046 | 0.025 | 1.861 | 0.066 |
|  |  |  |  |  |
| Call Duration |  |  |  |  |
| Soft-barrier condition | -0.042 | 0.034 | -1.224 | 0.351 |

| Short scream | Estimate | Std. Error | t value | P value |
| --- | --- | --- | --- | --- |
| Dominant Frequency |  |  |  |  |
| Soft-barrier condition | 0.019 | 0.035 | 0.577 | 0.583 |
|  |  |  |  |  |
| Call Duration |  |  |  |  |
| Soft-barrier condition | 0.039 | 0.043 | 0.922 | 0.359 |

| Squeak | Estimate | Std. Error | t value | P value |
| --- | --- | --- | --- | --- |
| Dominant Frequency |  |  |  |  |
| Soft-barrier condition | 0.068 | 0.032 | 2.17 | 0.038 |
|  |  |  |  |  |
| Call Duration |  |  |  |  |
| Soft-barrier condition | -0.023 | 0.054 | -0.425 | 0.679 |

| Twitter | Estimate | Std. Error | t value | P value |
| --- | --- | --- | --- | --- |
| Dominant Frequency |  |  |  |  |
| Soft-barrier condition | -0.007 | 0.039 | -0.177 | 0.861 |
|  |  |  |  |  |
| Call Duration |  |  |  |  |
| Soft-barrier condition | 0.025 | 0.073 | 0.338 | 0.737 |
